# Human pluripotent stem cell-derived alveolar epithelial cells are alternatives for *in vitro* pulmotoxicity assessment

**DOI:** 10.1038/s41598-018-37193-3

**Published:** 2019-01-24

**Authors:** Hye-Ryeon Heo, Jeeyoung Kim, Woo Jin Kim, Se-Ran Yang, Seon-Sook Han, Seong Joon Lee, Yoonki Hong, Seok-Ho Hong

**Affiliations:** 1 Department of Internal Medicine, School of Medicine, Kangwon National University, Chuncheon, 24341 South Korea; 2 Environmental Health Center, Kangwon National University Hospital, Chuncheon, 24341 South Korea; 3 Department of Thoracic & Cardiovascular Surgery, School of Medicine, Kangwon National University, Chuncheon, 24341 South Korea

## Abstract

Human pluripotent stem cell (hPSC)-derived alveolar epithelial cells (AECs) provide new opportunities for understanding lung development and the treatment of pulmonary diseases. However, toxicity assessments using hPSC-AECs have not been undertaken. In this study, we generated functional AECs from hPSCs and evaluated their inflammatory and apoptotic responses to cadmium (Cd) exposure (1, 5, and 10 μM) for 24 h compared with the human bronchial epithelial cell line (BEAS-2B) and primary AECs as controls. Our data showed that Cd (10 μM) treatment induced substantial inflammatory responses and apoptosis in BEAS-2B cells, but not in both hPSC-AECs and primary AECs. Interestingly, conditioned medium from AEC cultures significantly alleviated apoptotic and inflammatory responses to Cd exposure in BEAS-2B cells. Using cytokine arrays, several potential factors secreted from hPSC-AECs and primary AECs were detected and may be involved in reducing Cd-induced cytotoxicity. We also observed higher expression of surfactant proteins B and C in both hPSC-AECs and primary AECs, which may contribute to protection against Cd-induced cytotoxicity. These results suggested that hPSC-AECs phenotypically and functionally resemble primary AECs and could be more biologically relevant alternatives for evaluating the pathological contribution of confirmed or potential pulmotoxic materials included in smoking and microdust.

## Introduction

Microdust is an environmental risk factor for respiratory diseases as air pollution spreads worldwide^1^. Smoking is also widely accepted as a primary cause of diseases in the lung and other organs^2^. *In vitro* models using primary bronchial and alveolar epithelial cells (AECs) are the most appropriate cells for evaluating the cytotoxic effects of toxic components in microdust and smoking relevant to pulmonary diseases. However, primary cells derived from different donors can show distinct responses depending on genetic background, patient age, and the type of tissue source. In addition, the characteristics of primary cells may change due to multiple passages during *in vitro* cultivation^3,4^. Immortalized cell lines, such as normal bronchial epithelial (BEAS-2B) and lung adenocarcinoma (A549) cells, have been widely used instead of primary cells to evaluate the cytotoxicity of suspected harmful materials^5–8^. However, increasing evidence shows that BEAS-2B and A549 cells respond to toxins differently than primary cells, and their phenotypes and functions are altered by culture conditions^9^. Thus, use of biologically relevant sources to assess the harmful effects of environmental risk factors on the human respiratory tract is needed to understand how they contribute to pulmonary diseases.

Human pluripotent stem cells (hPSCs), including human embryonic stem cells (hESCs) and induced PSCs (iPSCs), can potentially generate an unlimited number of somatic cells that offer *in vitro* predictive models for evaluating environmental toxins and for large-scale screening of novel drugs as well as cell therapies^10^. Although reports are limited, several differentiated cell types derived from hPSCs may be useful for such toxicity testing. Neural progenitor cells derived from hESCs have been used to study the neurotoxic effects of lead and gold nanoparticles on early brain development^11^. The toxic effects of short- and long-term drug (amiodarone, aflatoxin B1, troglitazone, ximelagatran, and doxorubicin) exposure have been investigated in hepatocytes and cardiomyocytes derived from hiPSCs and hESCs^12,13^. Two independent research groups have developed three-dimensional spheroids as *in vitro* models using mature hepatocytes or neuronal precursors derived from hPSCs, and have demonstrated their applications for drug toxicity testing^14,15^. More recently, hepatotoxicity against the herbal medicines has been evaluated using hESC-derived hepatocytes, which showed similar toxicity patterns to human primary cultured hepatocytes^16^. All these reports indicated that hPSC derivatives have the potential to be used in cytotoxicity evaluations of various harmful materials and drugs, and could be alternatives for the replacement of cell lines and primary cells.

Recent studies reported the generation of functional AECs derived from hiPSCs and hESCs and their therapeutic applications for acute and chronic pulmonary diseases^17–21^. However, toxicity assessments using hPSC-AECs have not been undertaken. In this study, we presented the first investigation of cadmium (Cd) cytotoxicity in hiPSC-derived AECs and compared cellular responses, gene expressions, and secretomes using BEAS-2B cells and human primary AECs after Cd exposure.

## Results

### Generation of functional AECs from hiPSCs

**Figure 1:**
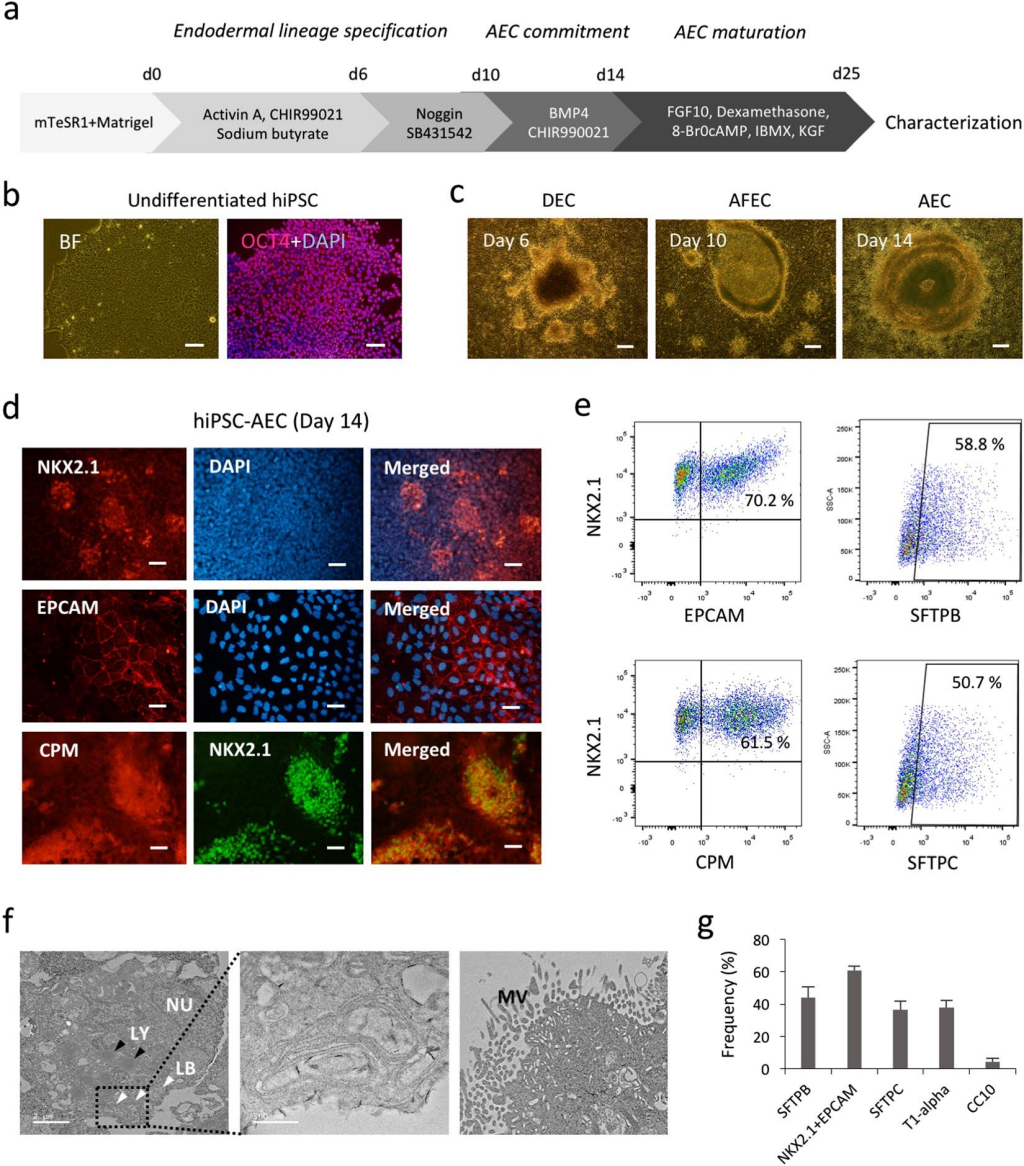
Generation and characterization of hiPSC-AECs. (**a**) Schematic diagram of stepwise AEC differentiation from hiPSCs based on lung developmental process. (**b**) Immunofluorescence staining for OCT4 (red) in feeder-free hiPSC cultures. Nuclei were counterstained with DAPI (blue). Scale bar: 100 μm. BF, bright field. (**c**) Representative images of AECs differentiated from hiPSCs. Scale bars: 500 μm. DEC, definitive endodermal cell; AFEC, anterior foregut endodermal cell. (**d**) Immunofluorescence staining for NKX2.1 (red or green), EPCAM (red) and CPM (red) in hiPSC-derived AECs on day 14 of differentiation. Scale bar: 100 μm. (**e**) Representative FACS plots based on expression of NKX2.1, EPCAM, CPM, STFPB and STFPC in hiPSC-AECs. Frequencies are shown in each plot. (**f**) TEM of day 14 hiPSC-AECs. Black arrowheads indicate lysosome (LY). White arrowheads indicate LBs. NU, nucleus. (**g**) Flow cytometry analysis of AEC1 and AEC2 marker expression in day 25 hiPSC-AECs. Error bars indicate SD.

To assess cellular responses after Cd exposure in hiPSC-AECs, BEAS-2B cells, and primary AECs, we performed alveolar epithelial specification, commitment, and maturation from undifferentiated hiPSCs using a sequential differentiation protocol mimicking the process of embryonic pulmonary development (Fig. 1a). Undifferentiated hiPSCs maintained chemically defined mTeSR1 serum-free medium showed strong expression of octamer-binding transcription factor 4 (OCT4), a marker for undifferentiated cells (Fig. 1b). As differentiation progressed, hiPSCs displayed significant morphological changes with polygonal and cuboidal epithelial-like shapes (Fig. 1c). In AEC commitment phase (from day 10 to day 14), the differentiated cells were strongly positive for alveolar epithelial progenitor markers including NKX2.1 (also known as thyroid transcription factor), epithelial cell adhesion molecule (EPCAM), and CPM (Fig. 1d). More than 90% of the cells were NKX2.1-positive, while the percentage of cells co-expressing NKX2.1 and EPCAM was approximately 70%. The cells also expressed the mature type 2 AEC (AEC2) markers such as SFTPB and SFTPC (58.8% and 50.7%, respectively) (Fig. 1e). SFTPB and SFTPC synthesized in endoplasmic reticulum of type 2 alveolar epithelial cells (AEC2) are stored in the lamellar body (LB) and secreted by exocytosis. Transmission electron microscopy (TEM) clearly showed the presence of LBs and microvilli (MV), typical of AEC2, indicating that functional AEC2 are efficiently generated during the AEC commitment phase (Fig. 1f). AEC2 have the potential to transdifferentiate into type 1 AECs (AEC1) during normal lung development or after lung injury. In the AEC maturation stage, the AEC2 differentiated to AEC1, which was determined by the expression of T1-alpha on day 25 of differentiation (Fig. 1g). We also found comparable numbers of lung mesenchymal cells expressing PDGFR-β, CD90, NG2, and CD146 on day 25 of differentiation (Supplementary Fig. 1). These observations suggest that our induction protocol efficiently generates functional AECs and related niche cells with phenotypes of alveolar epithelial and mesenchymal cells.

**Figure 2:**
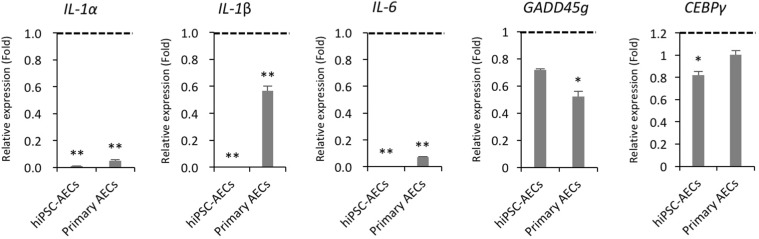
Comparison of the basal expression levels of inflammation- and apoptosis-related genes in BEAS-2B cells, hiPSC- and primary AECs. Quantitative real-time PCR analysis of inflammation and apoptosis-related gene expression in BEAS-2B cells, hiPSC- and primary AECs. Black dotted lines indicate transcript levels of genes in BEAS-2B cells. Error bars indicate SD. *p < 0.05, **p < 0.01 (BEAS-2B cells *vs*. hiPSC-AECs or primary AECs).

Controlling inflammatory and apoptotic responses in AECs during external toxin exposure is important for maintaining homeostatic lung functions and preventing pathological progression^22^. Thus, we compared the basal transcript levels of genes encoding proteins related to inflammation (*IL-1α, IL-1β, and IL-6*) and apoptosis (*GADD45g and CEBPγ*) in BEAS-2B cells, hiPSC-AECs (day 14), and primary AECs. Overall, the basal transcript levels of genes assessed in hiPSC-AECs and primary cells were lower than those in BEAS-2B cells (Fig. 2). Interestingly, hiPSC-AECs and primary AECs showed significantly lower transcript levels of *IL-1α, IL-1β, and IL-6* compared with BEAS-2B cells. We also found that the transcript level of *IL-1β* in hiPSC-AECs was extremely low compared with that in other cell types. Taken together, these results indicated that hiPSC-derived AECs phenotypically and morphologically resembled *in vivo* AECs and may be appropriate for lung cytotoxicity evaluations.

### hiPSC-derived cells and primary AECs were more resistant to Cd cytotoxicity than BEAS-2B cells

**Figure 3:**
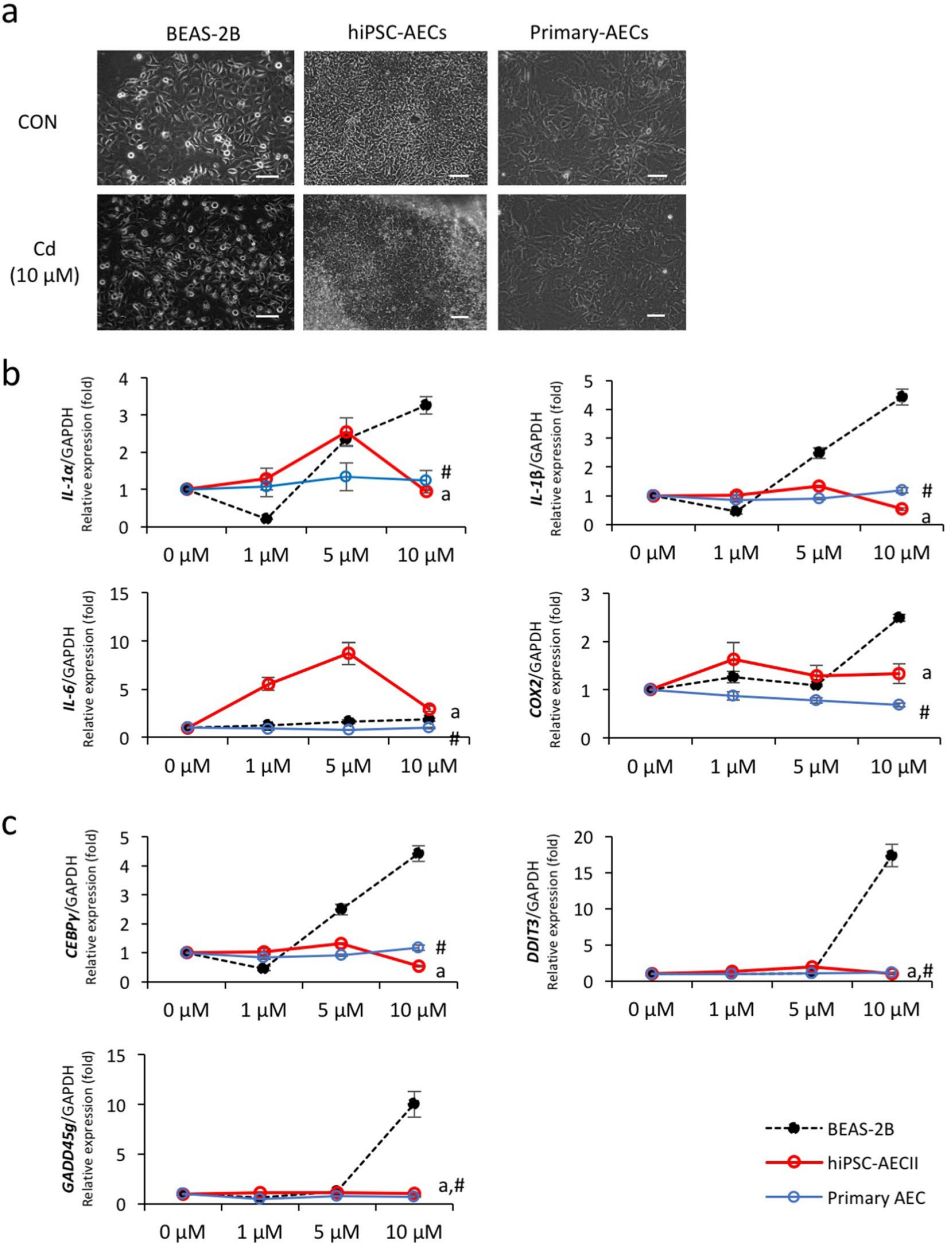
Effects of Cd exposure on inflammation- and apoptosis-related gene expression in BEAS-2B cells, hiPSC- and primary AECs. (**a**) Morphological changes in BEAS-2B cells, hiPSC- and primary AECs after Cd treatment. All cells were cultured in the absence or presence (10 μM) of Cd for 24 h. Scale bar: 100 μm. (**b**,**c**) BEAS-2B cells and primary AECs were treated with the indicated concentrations of Cd for 24 h. On day 14 of differentiation, AECs were treated with the same Cd doses. Transcript levels of inflammation- (**b**) and apoptosis-related genes (**c**) were measured using quantitative real-time PCR. Black dotted line, BEAS-2B; red line, hiPSC-AECs; blue line, primary AECs. Error bars indicate SD. ^a^*p* < 0.05 (BEAS-2B cells *vs*. hiPSC-AECs), ^#^*p* < 0.05 (BEAS-2B cells *vs*. primary AECs).

Using functional AECs derived from hiPSCs, we assessed if the cells could be an alternative resource for testing pulmotoxicity of environmental toxicants. We cultured hiPSC-AECs on day 14 of differentiation and BEAS-2B cells in the absence or presence of Cd and investigated their cellular responses compared with primary AECs. According to previous studies, Cd absorption in the lungs depends on the chemical nature of particles deposited^23^. Thus, we used soluble Cd form (cadmium chloride, CdCl_2_) for the greater toxic potency compared with insoluble Cd compounds such as Cd metal, Cd carbonate (CdCO_3_) and Cd selenide (CdSe). Dead cells floating on the medium and morphological changes from the typical epithelial shape to round cells were clearly observed after exposure to 10 μM Cd in BEAS-2B cells, but not in hiPSC-derived cells and primary AECs (Fig. 3a). We then compared alterations of genes related to inflammation and apoptosis in each cell type treated with Cd. Treatment with Cd gradually increased expressions of inflammatory-related genes (*IL-1α, IL-1β*, *IL-6*, and *COX2*) in a dose-dependent manner in BEAS-2B cells (Fig. 3b). However, their expressions in both hiPSC-derived cells and primary AECs were not significantly altered by Cd exposure, although transcript levels of *IL-1α* and *IL-6* in hiPSC-AECs increased after 1 and 5 μM Cd exposure. We further compared alterations of apoptosis-related genes (*CEBPγ*, *GADD45g*, and *DDIT3*) in each cell type after Cd treatment. Similarly, transcript levels of these genes were not changed after Cd (1, 5, and 10 μM) exposure in hiPSC-derived cells and primary AECs, but significantly increased in BEAS-2B cells upon exposure to 10 μM Cd (Fig. 3c). These results indicated that hiPSC-derived cells and primary AECs were more resistant to Cd cytotoxicity than BEAS-2B cells and were functionally similar to each other following Cd exposure.

### hiPSC-AECs alleviated Cd-induced cytotoxicity in BEAS-2B cells via paracrine mechanisms

**Figure 4:**
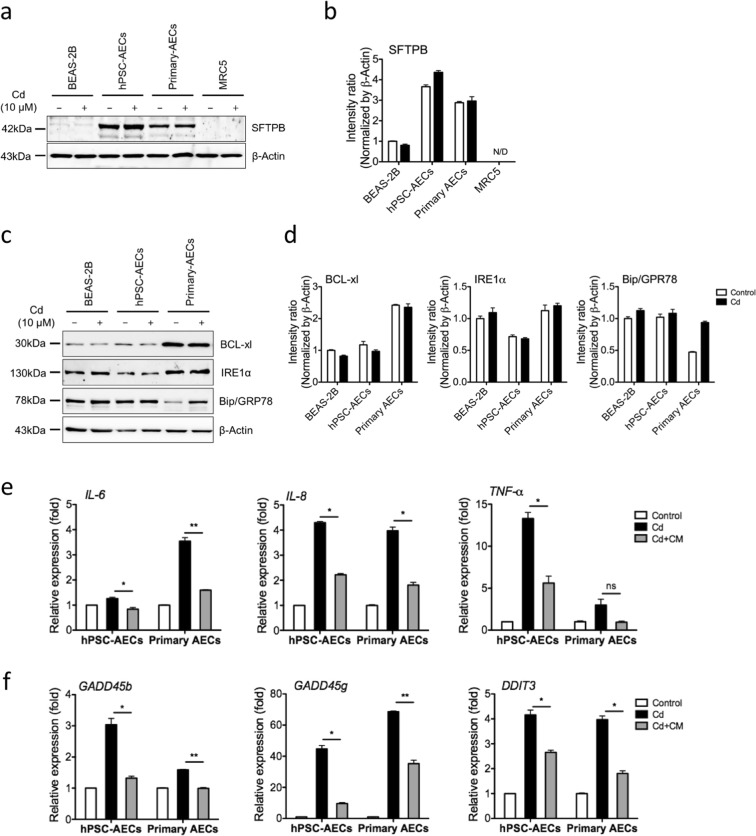
hiPSC-AECs attenuate Cd-induced cytotoxicity in BEAS-2B cells via paracrine effects. (**a,b**) All cells were cultured in the absence or presence (10 μM) Cd for 24 h. Cell lysates were extracted and subjected to Western blot analysis to determine protein levels of SFTPB. (**c,d**) Western blot analysis to determine protein levels of BCL-xl, IRE1α, and Bip/GRP78. (**e**,**f**) Transcript levels of inflammation-related (**e**) and apoptosis-related genes (**f**) in BEAS-2B cells exposed to Cd in the presence and absence of CM collected from hiPSC-AEC and primary AEC cultures. Full length blots are presented in Supplementary Fig. 2. Error bars indicate SD. *p < 0.05, **p < 0.01.

We assumed that hiPSC- and primary AECs had defense mechanisms against toxic materials. AECs are functionally characterized by the production of SFTPB, which form surface layers that coat alveoli conditioned by the air-liquid interface, prevent lung collapse, and aid in the maintenance of innate immunity^24^. These proteins also play an important role in recovery from lung injury due to toxic heavy metal exposure^25^. Thus, we examined the expression levels of SFTPB in all cell types cultured in the absence or presence (10 μM) of Cd. SFTPB was strongly expressed in both hiPSC-AECs and primary AECs, but was very weakly expressed or not detected in BEAS-2B and human adult lung fibroblast cell (MRC5) lines (Fig. 4a). The expression level of SFTPB was slightly increased only in Cd-treated hiPSC-AECs, but the increase did not reach statistical significance (Fig. 4b). These results suggest that hiPSC-AECs and primary AECs may provide a protective mechanism against Cd cytotoxicity via secretion of SFTPs. We also found that expression levels of anti-apoptotic BCL-xl and ER stress-related IRE1α were not different among the cell types, while Bip/GRP78 expression was increased in primary AECs (Fig. 4c,d). We then collected conditioned media (CM) from hiPSC-AEC and primary AEC cultures to determine if there were potential paracrine factors that could alleviate Cd-induced cytotoxicity in BEAS-2B cells. We found that Cd (10 μM)-induced inflammatory and apoptotic responses in BEAS-2B cells were significantly reduced by incubating with CM derived from both hiPSC-AEC and primary AEC cultures (Fig. 4e,f). These results indicated that hiPSC- and primary AECs attenuated Cd-induced cytotoxicity in human bronchial epithelial cells via paracrine mechanisms.

### Secretome analyses identified potential defense factors of hiPSC-AECs against toxic materials

**Figure 5:**
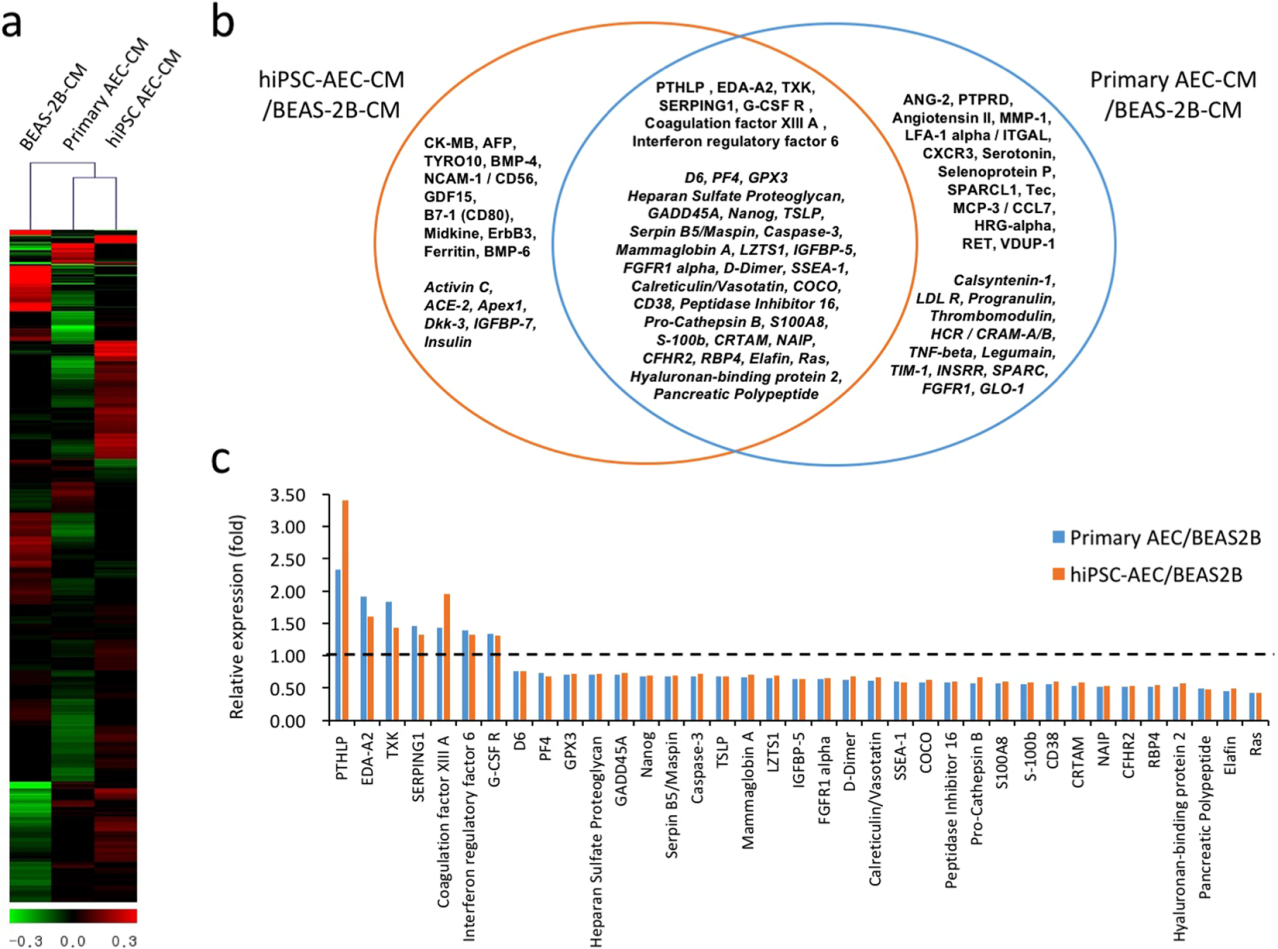
Secretome analysis in CMs harvested from hiPSC-AEC, primary AEC and BEAS-2B cell cultures. Heat maps for secretions of BEAS-2B, hiPSC- and primary AECs. The color spectrum from green to red represents low to high expression (**a**). Venn diagram shows commonly up- (in bold) and downregulated (in bold italics) proteins in CMs of hiPSC- and primary AECs compared to CM of BEAS-2B (**b**). Expression levels of commonly up- and down-regulated 37 secretions in CMs of hiPSC- and primary AECs (**c**). Black dotted line indicates expression levels of genes in BEAS-2B cells.

To determine if hiPSC-AECs secreted potential defense factors to protect against the cytotoxicity of Cd, we profiled cytokine secretions in CM harvested from hiPSC-AECs, primary AECs, and BEAS-2B cell cultures using a cytokine array consisting of 1,000 different cytokines and chemokines. We found that hiPSC-AECs showed a similar secretion profile as that of primary AECs, compared with BEAS-2B cells (Fig. 5a). Thirty-seven secreted factors (7 upregulated and 30 downregulated) were found to be present in CM from both primary cells and hiPSC-AECs (Fig. 5b,c). Using GO and KEGG pathway analyses, 37 common secreted factors were organized by biological processes and cellular components, indicating that the factors were correlated to each other in cytokine-cytokine and extracellular interactions (Table 1). Among the upregulated seven factors, ectodysplasin A2 (EDA-A2), serine proteinase inhibitor (Serpin G1), coagulation factor XIII A, and interferon regulatory factors (IRFs) are known to be associated with host defense against toxins or reversing a developmental disease in the lung. The factors from AEC CM may contribute to the maintenance of their basic functionalities and could provide a molecular basis to understand the defense mechanisms of hPSC-AECs and primary AECs against environmental toxic materials.

**Table 1:** Gene ontology (GO) analysis for secreting factors that commonly up- and downregulated in CMs of hiPSC- and primary AECs compared to CM of BEAS-2B cells.

Term name	p-value	Secretions
***GO Biological process***		
Negative regulation of endopeptidase activity	0.001416	SERPING1, Hyaluronan-binding protein 2, Elafin, Serpin B5/Maspin
Cytokine-mediated signaling pathway	0.001778	PF4, EDA-A2, Ras, G-CSF R
Response to glucocorticoid	0.006382	Caspase-3, Ras, S-100b
Positive regulation of cell proliferation	0.010178	FGFR1 alpha, PTHLP, Ras, S-100b, Calreticulin/Vasotatin
Female pregnancy	0.01169	CD38, PTHLP, IGFBP-5
Response to estradiol	0.012196	CD38, Calreticulin/Vasotatin, Caspase-3
Positive regulation of gene expression	0.012228	PF4, EDA-A2, Calreticulin/Vasotatin, Ras
Platelet degranulation	0.015432	PF4, SERPING1, Coagulation factor XIII A
Striated muscle cell differentiation	0.018313	IGFBP-5, Ras
Positive regulation of NF-kappa B transcription factor activity	0.024922	EDA-A2, S100A8, Ras
***GO Cellular component***		
Extracellular region	3E-10	PF4, RBP4, Hyaluronan-binding protein 2, FGFR1α, Calreticulin/Vasotatin, GPX3, Pro-Cathepsin B, Pancreatic Polypeptide, S100A8, Coagulation factor XIII A, CFHR2, EDA-A2, SERPING1, PTHLP, IGFBP-5, COCO, G-CSF R, S-100b
Extracellular space	3E-07	PF4, RBP4, Hyaluronan-binding protein 2, Calreticulin/Vasotatin, Pro-Cathepsin B, Pancreatic Polypeptide, GPX3, S100A8, TSLP, SERPING1, Serpin B5/Maspin, PTHLP, COCO, S-100b
Extracellular exosome	3E-03	Peptidase Inhibitor 16, NAIP, RBP4, Hyaluronan-binding protein 2, Elafin, Calreticulin/Vasotatin, Pro-Cathepsin B, GPX3, IRF6, S100A8, CD38, SERPING1, Serpin B5/Maspin
Platelet alpha granule lumen	4E-03	PF4, SERPING1, Coagulation factor XIII A
***KEGG pathway***		
Signaling pathway	0.01424	GADD45A, Serpin B5/Maspin, Caspase-3
Complement and coagulation cascades	0.015061	SERPING1, Hyaluronan-binding protein 2, Coagulation factor XIII A
Proteoglycans in cancer	0.016442	FGFR1α, Nanog, Caspase-3, Ras
Cytokine-cytokine receptor interaction	0.023784	PF4, EDA-A2, TSLP, G-CSF R
MAPK signaling pathway	0.031078	GADD45A, FGFR1α, Caspase-3, Ras

## Discussion

Cd, a major component of both microdust and cigarettes, disrupts homeostatic normal lung functions. Numerous studies have used lung epithelial cell lines, such as BEAS-2B and A549 cells, to predict respiratory disease-associated Cd cytotoxicity. For example, subtoxic Cd doses increased the expression of extracellular matrix molecules in BEAS-2B cells mediated by TGF-β signaling^26^. Acute doses of Cd also disrupted tight junction integrity and reduced the cystic fibrosis transmembrane conductance regulator expression in BEAS-2B cells via kinase activation^27,28^. Chen *et al*. reported that Cd caused oxidative stress-induced cytotoxicity in BEAS-2B cells, after they performed a proteomics analysis of secreted proteins in response to Cd exposure^29^. Cd also affected transcript levels of inflammation-related cytokines in A549 cells, indicating that anti-inflammatory drugs may prevent Cd-induced cytotoxicity^30^. Although these findings suggested a causative role of Cd in the development of pulmonary diseases, such research can yield unreliable results due to genetic and functional discrepancies between cell lines and primary AECs^4^, and due to the limited feasibility of toxicant testing in human subjects. Recently, the differentiation capability of hiPSCs has been recognized as an *in vitro* predictive model for cytotoxicity testing of environmental toxicants and for regenerative medicine research. Several differentiated cell types derived from hiPSCs, including cardiomyocytes, hepatocytes, and neural progenitors, have been tested for short- and long-term adverse effects of drugs or environmental toxicants. However, toxicity assessment using hiPSC-derived AECs has not been undertaken. In the present study, we generated functional AECs from hiPSCs and provided the first report comparing their phenotypes, differential gene expressions, and cellular responses to Cd exposure using BEAS-2B cells and primary AECs. Our findings indicated that hiPSC-AECs differed from other cell lines but displayed similar phenotypes, gene expression patterns, and cellular responses to Cd exposure as those of primary AECs. Thus, hiPSC-AECs are more biologically useful to evaluate the cytotoxicity of environmental toxicants associated with pulmonary diseases than the cell lines previously utilized.

The lungs are vulnerable to pathogens and harmful substances due to their continuous exposure to environmental toxicants through inhalation. However, if the lung mechanisms sensitively counteracted every external stimulus, excessive inflammation and apoptosis could lead to damage and secondary pathology after these reactions occurred. Thus, the lungs appropriately adapt to the environment by maintaining homeostatic equilibrium, which is partially achieved by surfactants that are membrane-based lipid-protein complexes that effectively exchange air and serve as barriers against environmental insults^25^. Pulmonary surfactants also play a major role in lowering surface tension at the air-water interface to prevent alveolar collapse and in maintaining immune quiescence in the lungs^31^. It has been reported that sustainable expression of SFTPB, a surfactant component, increased survival of the distal lung epithelium during nickel-induced acute lung injury^32,33^. Carbon nanotubes (CNTs) promise a wide range of applications in biomedical sciences as well as in electric engineering and industry. However, the adsorption of pulmonary surfactants on inhaled CNTs enhances recognition of the CNTs by alveolar macrophages, leading to increased inflammatory responses, oxidative stress, and fibrotic changes^34,35^. In addition, patients with ARDS that received intrabronchial SFTP treatment showed extensive alterations in biochemical properties of SFTPs and improvement of alveolar surface activity^36^. These findings support our speculation that SFTPs contribute to gene expression stability and protect cells from Cd in conjunction with surfactant lipids. Indeed, SFTPB was detected in both hiPSC-derived and primary AECs, but not in BEAS-2B cells, and may serve as the frontline defense system of AECs against Cd cytotoxicity.

In the lungs, there are various protectors against toxins or inducers of repair as well as surfactant proteins. In this study, we showed that treatment with hiPSC-AEC-CM alleviated Cd-induced cytotoxicity in BEAS-2B cells, which might be mediated by paracrine effects. Cytokine arrays revealed that hiPSC-AEC-CM was abundant in cytokines and chemokines, among which EDA-A2, Serpin G1, coagulation factor ΧIII A, and IRF6 levels were high. Genetic defects in the *EDA* gene resulted in frequent respiratory infections due to the absence of tracheal and bronchial glands. The administration of recombinant EDA achieved significant correction of the missing tracheal and bronchial glands^37^. Serpin G1 has anti-histone properties that protect against lung injury by histone-induced cell death^38^. Coagulation factor ΧIII A has a positive effect on repairing organ injury in rats after trauma-hemorrhagic shock by reducing lung permeability, neutrophil sequestration, oxidative stress, and pro-inflammatory cytokines, as well as increasing anti-inflammatory cytokines such IL-10^39^. In addition, the IRF family contains various subtypes, of which IRF3 is associated with host defenses against viral infections. Specially, IRF3 contributes to the clearance of *Pseudomonas aeruginosa* from the lungs of mice^40^. All these previous findings suggested that potential defense mechanisms of these secreted factors contributed to the resistance of hPSC-AECs and primary AECs against Cd exposure.

As air pollution increases, studies of lung pathologies induced by various toxicants are essential for preventing certain respiratory diseases by predicting the substance’s cytotoxicity. In our study, we assessed the reliability of cytotoxicity testing in functional AECs derived from hiPSCs. Additionally, we showed the possibility that some of the secreted factors derived from hiPSC-AECs might be used as protection against toxins, or as inducers of lung repair. In the future, more detailed comparative studies of primary AECs and related cell lines should be performed to alleviate the toxicity of fine dusts and various heavy metals. Further comparisons of genetic and epigenetic changes in both hiPSC-derived and primary AECs due to toxic substance exposure may also enable the treatment of certain respiratory diseases and could aid in biomarker discovery.

## Methods

### Cell cultures

Human iPSCs (iPS-NT4-S1) were kindly provided by CHA University, Seoul, South Korea. The cells were maintained in mTeSR1 serum-free medium (Stem Cell Technologies, Canada) on Matrigel (BD Bioscience, USA)-coated dishes. They were subcultured at 80% confluency and passaged every 5 days by mechanical dissociation. BEAS-2B cells were obtained from Seoul National University Hospital and were cultured in defined keratinocyte serum-free medium (Gibco, USA) supplemented with epidermal growth factor and 1 U penicillin/streptomycin. Primary AECs were purchased from ScienCell Research Laboratories and were cultured in alveolar epithelial cell medium (ScienCell, USA) containing epithelial cell growth supplement and 10 ml fetal bovine serum (Hyclone, USA) on dishes coated with poly-L-lysine (Sigma, USA). All cells were incubated at 37 °C in a humidified atmosphere with 5% CO_2_.

### Stepwise differentiation of hiPSCs into AECs

Multistep AEC differentiation was performed as previously described with some minor modifications^17^. Briefly, undifferentiated hiPSC colonies were prepared with low density of less than 5 colonies per well. When the colonies grew to approximately 1 mm in diameter, AEC differentiation was initiated with exposure to sequential induction medium and was assessed by observing the localization and frequencies of AEC-specific markers on Day 14 post-initiation using immunostaining and flow cytometry. On day 15 of differentiation, AECs were washed with phosphate buffered saline (PBS) and replenished with serum-free induction medium for 24 h prior to harvesting the media for further experiments. Conditioned medium (CM) was centrifuged at 1,500 rpm for 4 min to remove cell debris and filtered through a 0.22 µm filter. Then, hiPSC-AECs were kept at −80 °C until use.

### Immunofluorescence staining

Undifferentiated hiPSCs and AECs were rinsed with PBS and fixed with 4% paraformaldehyde (Alfa Aesar) for 20 min at room temperature. Cells were permeabilized with 0.5% saponin (Sigma) and blocked with 10% normal donkey serum (Jackson Immuno., USA) diluted with 1% BSA in PBS. The following primary and secondary antibodies were used: rabbit anti-OCT4 (1:200, BD Pharmingen), rabbit anti-NKX2.1 (1:250, Abcam), mouse anti-CPM (1:500, Abcam), mouse anti-EPCAM (1:400, Santa Cruz), Alexa 594 (Invitrogen) donkey anti-mouse IgG(H + L), Alexa 488 (Invitrogen) donkey anti-rabbit IgG(H + L) and Alexa 594 (Invitrogen) donkey anti-rabbit IgG(H + L). Nuclei were counterstained with Fluoroshield with DAPI (Sigma) for 5 min, and fluorescent images were captured with a fluorescence microscope (IX-51, Olympus, JAPAN).

### Transmission electron microscopy

Human iPSC-AECs were processed as previously described^41^. The cells were fixed for 3 h in 0.1 M cacodylate buffer (pH 7.4) containing 4% glutaraldehyde and 1% paraformaldehyde. After three washes in 0.1 M cacodylate buffer, the cells were dehydrated through a gradual gradient series of ethanol, 20 min each step, starting from 50% ethanol and ending with 100% ethanol. The cells were incubated with an increasing gradient of propylene oxide in ethanol and then infiltrated with an increasing concentration of Eponate 812 resin in ethanol. Samples were baked at 65 °C for 24 h and then sectioned using an Ultra microtome. Sections were observed under a Field Emission Transmission Electron Microscope (JEM-2100F, Japan) at the Korean Basic Science Institute (Chuncheon), South Korea.

### Exposure of cell cultures to Cd

BEAS-2B cells (passage 14) were plated into 35 mm dish (3 × 10^5^ cells per 10 mm^2^ area of dishes). Cryopreserved primary AECs (passage 0) were rapidly thawed in a 37 °C water bath (<1 min) and seeded into T-75 flask (>5 × 10^5^ cells in 1 vial). Then, the cells were allowed to attach overnight and replenished with a fresh medium the next morning. Once the cells reached 90% confluency, the cells were subcultured into 6-well plate and maintained for 2 days. Then, BEAS-2B (passage 15) and primary AECs (passage 1) were changed to a fresh medium and kept for 24 h prior to Cd treatment with various concentrations (0, 1, 5 and 10 μM). On day 14 of AEC differentiation, hiPSC-AECs were rinsed with PBS and replenished with induction medium for 24 h prior to Cd treatment. CdCl_2_ (Sigma, Cat no. 202908) was dissolved in distilled water to make a stock of 10 mM solution, which was added directly to the culture medium to give final concentrations of 1, 5 and 10 μM. The cells were cultured in the presence and absence of Cd for 24 h and collected for further analysis.

### Flow cytometry

Differentiated hiPSC-AECs were incubated with collagenase IV (Sigma) for 2 h, followed by treatment with cell dissociation medium (Gibco) for 20 min at 37 °C. Cells were passed through a 70 μm cell strainer and incubated with primary antibodies targeting the following molecules: NKX2.1 (Abcam, UK), carboxypeptidase M (CPM, Abcam), epithelial cell adhesion molecule (EPCAM, Santa Cruz), Surfactant protein B (SFTPB, EMD Millipore, USA), SFTPC (Abcam), T1α/Podoplanin (Abcam) and CC10 (Santa Cruz). Dead cells were excluded based on staining with 7-aminoactinomycin D (BD Pharmingen). Frequencies of AEC markers were measured using a FACSCanto^TM^ II flow cytometer (BD Bioscience), and acquired data were analyzed with FlowJo software (Tree Star).

### Quantitative real-time PCR

Total RNA was extracted from cells using the RNeasy Kit (Qiagen, USA), and cDNA was synthesized from 1 μg total RNA using the TOPscript^TM^ RT DryMIX kit (RT200, Enzynomics, Korea). Transcripts were quantified using TOPreal^TM^ qPCR 2X PreMIX (RT501S, Enzynomics) on the QuantStudio^TM^ 6 Flex Real-Time PCR system (Applied Biosystems, USA). Data were normalized to GAPDH expression, and sequences of all primers used are listed in Table 2.

**Table 2:** Primer sequences used in quantitative real-time PCR.

Gene		Sequence
*IL-1α*	F R	ATCAGTACCTCACGGCTGCT TGGGTATCTCAGGCATCTCC
*IL-1β*	F R	CTGTCCTGCGTGTTGAAAGA TTCTGCTTGAGAGGTGCTGA
*IL-6*	F R	TACCCCCAGGAGAAGATTCC TTTTCTGCCAGTGCCTCTTT
*IL-8*	F R	GTGCAGTTTTGCCAAGGAGT CTCTGCACCCAGTTTTCCTT
*COX2*	F R	TGCTTGTCTGGAACAACTGC TGAGCATCTACGGTTTGCTG
*TNF-α*	F R	AACCTCCTCTCTGCCATCAA CCAAAGTAGACCTGCCCAGA
*GADD45b*	F R	TGCTGTGACAACGACATCAAC GTGAGGGTTCGTGACCAGG
*GADD45g*	F R	CAGATCCATTTTACGCTGATCCA TCCTCGCAAAACAGGCTGAG
*CEBPγ*	F R	GAAAAAGAGCCGGTTGAAAAGC ACTGTACGTTGTCTGCAAGGT
*DDIT3*	F R	GGAAACAGAGTGGTCATTCCC CTGCTTGAGCCGTTCATTCTC
*GAPDH*	F	TGCACCACCAACTGCTTAGC
	R	GGCATGGACTGTGGTCATGAG

### Immunoblots

Cells were rinsed with cold PBS and lysed with Triton-X lysis buffer. Lysates were separated on a 10 or 12% SDS-PAGE gel and transferred onto a polyvinyldifluoride membrane. Membranes were probed with primary antibodies against anti-human SFTPB (EMD Millipore), BCL-xl (Santa Cruz), IRE1α, Bip/GRP78 (Cell Signaling Technology) and β-actin (Santa Cruz) overnight at 4 °C, followed by incubation with HRP-conjugated secondary anti-sera (Sigma). Power-Opti ECL^TM^ solution (Bionote, Korea) and a cooled CCD camera system (Bio-Rad Laboratories, Inc., USA) were used for visualizing the immunoblots.

### Cytokine array and data acquisition

CMs were harvested from BEAS-2B, primary AECs and hiPSC-AEC cultures for secretome anaylsis. The concentration of CMs was measured with BCA protein assay kit (Pierce, Rockford, I11) using Multi-Skan FC (Thermo, USA). The purities of purified CMs were confirmed on UV spectrum. The Human L507 Array slides (RayBiotech, Norcross, GA) were dried for 2 hr at room temperature and incubated 400 μl of blocking solution at room temperature for 30 min. After decanting the blocking buffer from each sub array, 400 μl of diluted CM was added and incubated for 2 hr at room temperature. After decanting the samples, all arrays were washed 3 times with 800 μl of 1X wash buffer I and incubated with biotin-conjugated anti-cytokine antibodies for 2 hrs. Then, the slides were incubated with Cy3-conjugated streptavidin solution and rinsed with de-ionized water. The result was scanned using GenePix 4100 A Scanner (Axon Instrument, USA) and analyzed with GenePix Pro 7.0 program (Axon Instrument, USA). Global normalization was conducted in the fluorescent spots to reduce noise and variation among the samples.

### Statistical analysis

Results are presented as means ± SD. Statistical comparisons between groups were conducted using the Student’s *t*-test with *p* < 0.05 as the cutoff for statistical significance.

## Supplementary information

Supplementary information
