# Supplementary material for: Human pluripotent stem cell-derived alveolar epithelial cells are alternatives for in vitro pulmotoxicity assessment
Source: Sci Rep. 2019 Jan 24;9:505. doi: 10.1038/s41598-018-37193-3 (PMC6346100; doi:10.1038/s41598-018-37193-3)
Supplement: Supplementary file 1 — Supplementary information [file 41598_2018_37193_MOESM1_ESM.docx]

**Human pluripotent stem cell-derived alveolar epithelial cells are alternatives for in vitro pulmotoxicity assessment**

Hye-Ryeon Heo^1,2#^, Jeeyoung Kim^1,2#^, Woo Jin Kim^1,2^, Se-Ran Yang^3^, Seon-Sook Han^1^, Seong Joon Lee^1^, Yoonki Hong^1,2*^, Seok-Ho Hong^1,2*^

^1^Department of Internal Medicine, School of Medicine, Kangwon National University, Chuncheon 24341, South Korea

^2^Environmental Health Center, Kangwon National University Hospital, Chuncheon 24341, South Korea

^3^Department of Thoracic & Cardiovascular Surgery, School of Medicine, Kangwon National University, Chuncheon 24341, South Korea

**
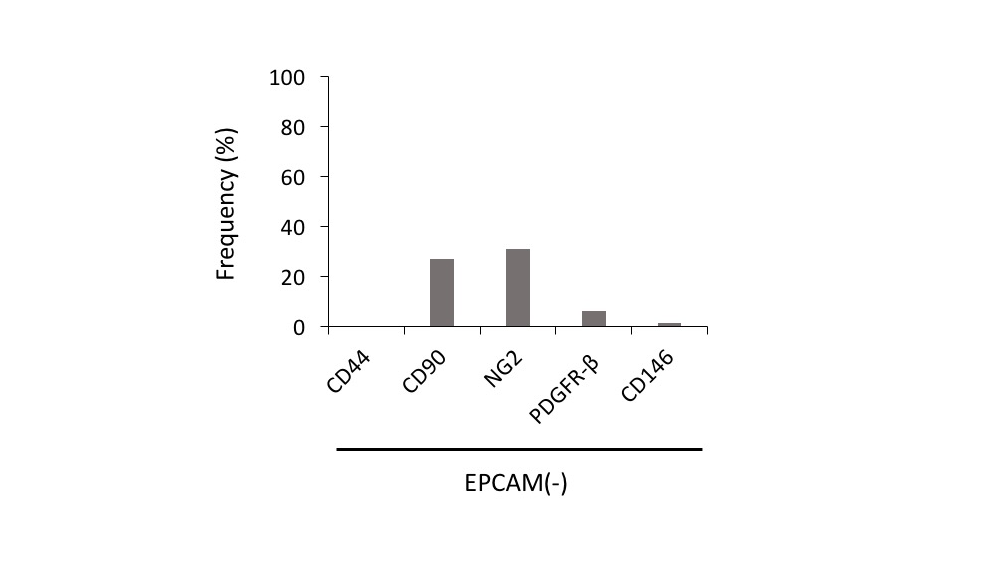
**

**Supplementary Figure 1. Expression of mesenchyme-related proteins in hiPSC-AEC cultures.** Flow cytometry analysis of mesenchyme cell markers in day 25 hiPSC-AECs. CD44 and CD90 (mesenchymal cell markers). NG2, PBGFR-β and CD146 (pericytes and smooth muscle cell markers).

**
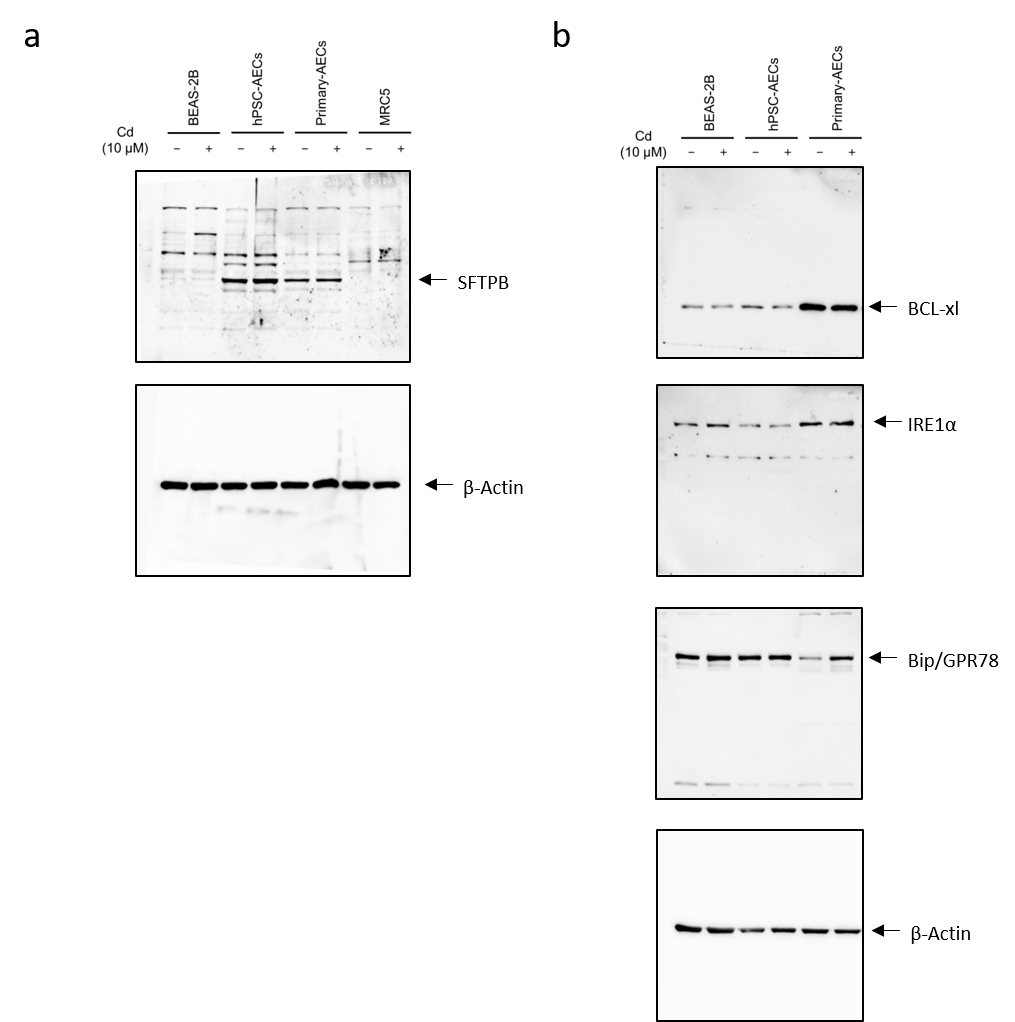
**

**Supplementary Figure 2. Western blot analysis for SFTPB/C, BCL-xl, IRE1α and Bip/GRP78 in BEAS-2B cells and AECs.** All cells were cultured in the absence or presence (10 μM) of Cd for 24 h. (**a**) Cell lysates were extracted and subjected to Western blot analysis to determine protein levels of SFTPB. (**b**) Apoptosis-related proteins including BCL-xl, IRE1α and Bip/GRP78. These full-length blots were cropped and slightly modified to black and white images of Fig. 4a and 4c.
